# Stem Cells as a Source of Pancreatic Cells for Production of 3D Bioprinted Bionic Pancreas in the Treatment of Type 1 Diabetes

**DOI:** 10.3390/cells10061544

**Published:** 2021-06-18

**Authors:** Michał Wszoła, Daria Nitarska, Piotr Cywoniuk, Magdalena Gomółka, Marta Klak

**Affiliations:** 1 Foundation of Research and Science Development, 01-793 Warsaw, Poland; michal.wszola@fundacjabirn.pl (M.W.); piotr.cywoniuk@gmail.com (P.C.); magdalena.gomolka.158@gmail.com (M.G.); 2 Polbionica Ltd., 01-793 Warsaw, Poland; daria.nitarska@polbionica.com; 3 Medispace Medical Centre, 01-044 Warsaw, Poland

**Keywords:** stem cells, pancreas, 3D bioprinting, diabetes, CRISPR/Cas9, alpha cells, beta cells

## Abstract

Type 1 diabetes (T1D) is the third most common autoimmune disease which develops due to genetic and environmental risk factors. Often, intensive insulin therapy is insufficient, and patients require a pancreas or pancreatic islets transplant. However, both solutions are associated with many possible complications, including graft rejection. The best approach seems to be a donor-independent T1D treatment strategy based on human stem cells cultured in vitro and differentiated into insulin and glucagon-producing cells (β and α cells, respectively). Both types of cells can then be incorporated into the bio-ink used for 3D printing of the bionic pancreas, which can be transplanted into T1D patients to restore glucose homeostasis. The aim of this review is to summarize current knowledge about stem cells sources and their transformation into key pancreatic cells. Last, but not least, we comment on possible solutions of post-transplant immune response triggered stem cell-derived pancreatic cells and their potential control mechanisms.

## 1. Introduction

The first successful isolated and cultured mouse (1981) [1,2] and human (1998) [3] embryonic stem cells (mESC, hESC, respectively) were milestones in the field of cell culturing, tissue engineering, disease modeling, and other fields of life sciences [4]. Stem cells were characterized with a potential to transform into any particular cell type of all three germ layers (ectoderm, endoderm, or mesoderm) and high proliferation rate which makes them, theoretically, an unlimited source of cells of any type [5,6,7]. When properly stimulated, stem cells have the ability to create particular tissue, including tumors and tissues disrupted by a genetic abnormality (i.e., when derived from patients suffering from a particular disorder [6,8]), or even clone the whole organism [9]. Such a groundbreaking discovery has elevated biological sciences to a significantly advanced level by creating opportunities to control the cell fate and culture cellular structures of higher level. On the other hand, such knowledge has raised serious ethical concerns about the embryonic source of stem cells and the ability to manipulate new life. Since then, numerous works have been published which describe new stem cell sources, precise protocols to create them and influence their fate, and present stem cell-based results of high value [10,11,12,13,14,15]. Application of stem cells is also thought to be a reasonable and promising pathway in seeking efficient therapy for Type 1 diabetes (T1D) [16,17,18,19], an immune-auto-aggressive disorder resulting in the destruction of pancreatic insulin-producing β-cells followed by glucose imbalance and its systemic repercussions [20]. The aim of this review is to summarize current knowledge about stem cell sources and their transformation into key pancreatic cells. Last, but not least, we comment on possible solutions of post-transplant immune response triggered stem cell-derived pancreatic cells and their potential control mechanisms.

## 2. Structure of the Pancreas and the Role of Individual Cells

The pancreas is an essential organ for the proper metabolism of nutrients and has both an endocrine and an exocrine function [21]. The exocrine part of the pancreas is made up of cells that secrete digestive enzymes and make up about 98% of the adult organ. Within the parenchyma are the islets of Langerhans that contain endocrine cells that produce hormones that play a key role in the maintenance of glucose homeostasis in the body. Each islet is a micro-organ that contains at least 4 cell types, including β (insulin), α (glucagon), δ (somatostatin), ε (ghrelin), and PP (pancreatic polypeptide) cells [22]. Type 1 diabetes (T1D) is one of the many serious diseases caused by disturbances in the functioning of the pancreas. It is one of the most common chronic diseases of the developmental age [20]. This heterogeneous disorder is characterized by the destruction of β cells, resulting in a complete insulin deficiency. In this type of diabetes there are 2 additional types: (a) autoimmune β-cell destruction, and (b) diabetes mellitus being idiopathic β cell destruction or failure [23,24]. T1D accounts for 5% to 10% of all diabetes cases worldwide. It is a chronic disease that causes many deaths every year. The susceptibility and resistance to T1D are caused by, among other things, the human HLA antigen complex, which is located on chromosome 6—primarily HLA class II [25]. Apart from genetic factors, age and sex, the so-called environmental risk factors are more and more often mentioned, acting early in life on genetically susceptible people, may trigger adverse immune processes [24,26].

## 3. Available Treatments for Patients with Type 1 Diabetes

For patients with Type 1 diabetes, life-saving treatment is multiple blood glucose control and administration of fast or slow-acting insulin. Glycemic control is currently the basic and best (though burdensome) treatment for patients, and it often affects their quality of life [24]. In the mid-1990s, insulin pumps became commonplace. They are small devices that enable continuous, subcutaneous infusion of insulin. The introduction of this type of device has eliminated the need for frequent punctures [27]. Equally important in the treatment of Type 1 diabetes is adequate physical activity and proper nutrition. It has been shown that a healthy lifestyle and frequent glycemic control significantly postpone the occurrence of life-threatening diabetic complications. However, despite increasing awareness, these complications are still the leading cause of death. Major complications include hyperglycemia, nephropathy, retinopathy, cardiovascular disease (CVD), and neuropathy [27,28,29,30]. In patients with diabetic autonomic neuropathy, life-threatening hypoglycemia can occur as a result of an impaired ability to recognize hypoglycemia. In fact, 6% of all deaths in patients with diabetes are due to severe hypoglycemia [31], especially at night. The only successful treatment for patients with the secondary complications of T1D is the transplantation of a whole pancreas or isolated pancreatic islets. Although pancreas transplantation can produce good long-term results in some patients, this treatment carries a risk of serious postoperative complications, such as acute rejection of graft, infections, or bacteremia [32,33,34]. Pancreas transplantation may be more appropriate for younger patients with fewer CVD complications. Those with a higher rate of CVD should rather be qualified for clinical islets transplantation (CITx) but this procedure is at a crossroads. Since the introduction of the Edmonton protocol [35], according to which islets often derived from two or three fresh islets preparations (around 13,000 islets equivalents (IE)/kg recipient body weight) are infused into the patient’s portal vein and special immunosuppression in which avoidance of corticosteroids and application of potent immunosuppression is very relevant, the improvement in islets survival was noticed. Nevertheless, improving long-term results is hard to achieve, even for the researchers who developed the protocol [36]. It is mainly due to islets apoptosis because of lack of extracellular matrix and lack of specific vasculature which is destroyed during the process of islets isolation. Another difficulty is an instant blood-mediated reaction observed after transplantation into a portal vein which diminishes the number of living islets. Developing new implementation sites, such as gastric submucosa [37,38,39], led to some improvement but could not solve a problem. There is a need to develop a technique as efficient as pancreas transplantation but with a complication rate close to the CITx procedure, thus to produce an organ with extracellular matrix and vasculature around islets, but one which is not complicated like in the native pancreas. Another important goal is to find a source of islets (α- and β-cells at least) for transplantation excluding the use of donors’ organs, preferably with patients’ own cells. It could also help in reducing or even omitting the need for an immunosuppressive treatment intake. Much interest is involved in stem cell transformation as a potential source of α- and β-cells for the treatment of diabetes. Moreover, observing the current progress in the development of medicine and sciences in the field of biotechnology, tissue engineering, and cellular transformation, the transformation of stem cells taken from the patient and their differentiation into insulin and glucagon producing cells seems a reasonable and very promising approach. Additionally, stem cells with ablation of HLA complex gain more and more interest as they give the possibility to construct universal cell lines which, can be applied for any patient without the need for immunosuppression [40].

## 4. Embryonic Stem Cells (ESC)

Stem cells derived from the inner cell mass of blastocyst isolated and cultured for the first time from a mouse in the early 80’ [1,2] through non-human primates [41] to successful isolation of hESC line in 1998 [3]. They characterize by high self-renewal potency and grow in tightly packed colonies in culture [42]. ESCs can be distinguished by the specific expression profiles of transcription factors (inhibiting activity of repertoire of the genes driving differentiation pathways) including SSEA-3, SSEA-4, TRA-1-60, TRA-1-81, Oct 3/4, and Nanog with simultaneous lack of SSEA-1 [42]. ESCs have paved the way in developmental biology, tissue engineering, cellular, and animal–human disease modeling, and regenerative medicine. However, while ESCs derived from animal models are widely used in basic research without major concerns, hESCs’ application raises ethical issues due to the human origin [43,44,45]. Much has been done since 1998 to improve the techniques of obtaining hESCs and their implementation in clinical procedures. First of all, a blastocyst derivation protocol has been upgraded which allows obtaining a single ESC from 8-cell blastomere, an earlier developmental stage of the blastocyst, without damaging the blastomere [46]. As ESCs require specific growing conditions including co-culturing with feeder cells, originally mouse embryonic fibroblasts (MEF) have been used. That, in turn, limited translation of isolated hESCs to clinical procedures because of the risk of xenotic antigens transmission. Currently, MEFs have been replaced with human feeder cell lines and feeder-free chemically supplemented culturing media [47,48,49,50]. Yet, the fact that hESCs are obtained during in vitro fertilization (IVF) procedure which does not meet the acceptance of part of the society is impossible to bypass. Another obstacle is the embryonic origin of hESCs. As in the vast majority of cases, hESCs cannot be acquired from the patient (it could be possible only for patients born by IVF) they can be transplanted only as an allograft which implicates the host’s immune system suppression.

## 5. Adult Stem Cells

Populations of cells retaining pluri- or multipotency found in adult tissues or organs such as bone marrow [51], olfactory mucosa [52], or mammary gland [53]. They reside in specific niches with microenvironment allowing them to keep undifferentiated state and replace damaged or dying specialized cells of the particular tissue [54]. One of the most ubiquitous and exploited adult stem cells are mesenchymal stem cells (MSC), spindle-shaped cells taken initially as fibroblasts [55] with specific surface protein markers set expressed (CD73^+^, 90^+^, 105^+^, CD14^−^, 34^−^, 45^−^) [56] occurring, among others, in adipose tissue [57], Wharton’s jelly, umbilical cord, or dental pulp. As mentioned, tissues treated as medical waste during procedures (liposuction, birth delivery, and dental extraction, respectively), they are attractive sources of easily accessible MSCs, and the ways of acquisition cause no ethical conflicts. What is more, it is possible to obtain MSCs directly from the patient, hence, re-transplanted cells will exhibit full histocompatibility. MSCs are capable of differentiating into chondrocytes, osteoblasts, neurons, myocytes, and cardiomyocytes, hepatocytes, and adipocytes, therefore, their application is limited [58]. Nonetheless, this differentiation spectrum is broad enough to place the MSCs in the spotlight of regenerative medicine. Recent works suggest MSCs’ role in tissue regeneration by immune system modulation and stimulation of angiogenesis [59,60,61,62,63,64]. Several others showed their utility after ex vivo propagation and nascent differentiation, as well as in situ and systemic injection in vivo. Simultaneously, works on broader use, including MSC-derived β-cells, are ongoing [65,66].

Currently, three approaches how MSCs could be applied to treat T1D are tested. The first approach is the use of MSC-derived cells which will be able to produce insulin and restore normoglycemia. There are a few reports which show that insulin-producing cells could be derived from MSCs [65,66,67,68,69,70,71]. Nevertheless, it is likely that the results presented in those studies are biased by the fact that only insulin concentration was evaluated while C-peptide was not determined. Only in the study performed by Prabakar et al. 2012, the level of the C-peptide was elevated after glucose stimulation in vitro [65]. The most challenging problem is related to the functionality of obtained cells. Thus, they are capable of insulin secretion, show expression of pancreatic transcription factors like PDX1, NEUROD1, NKX6.1 but they are not fully mature β-cells, so their ability to restore normoglycemia is limited. The second idea is to use undifferentiated MSCs to generate β-cells through direct trans-differentiation in vivo after transplantation, but this approach has scarcely been studied [72,73,74]. Despite that, two clinical trials where MSC-derived pancreatic progenitors generated in vitro maturated into β-cells after transplantation took place [75,76]. The outcome was quite promising. An increase in the amount of the C-peptide in serum and improved HbA1c values were obtained. The third approach how to use MSCs in T1D treatment which is currently the most often evaluated is to use undifferentiated MSCs to support islets health and survival [77,78,79,80,81,82]. There are a few potential mechanisms by which MSCs could work like reduction in inflammation, secretion of growth factors, and protection against hypoxia [83,84,85].

There is a premise that adult stem cells could be obtained from the pancreas. It was noticed that pancreatic exocrine cells like duct epithelial cells and acinar cells have differentiation potential and could be regarded as pancreatic progenitor cells (PPC). Trans-differentiation of ductal or acinar cells could be a potential source of β-cells for T1D treatment. Trans-differentiation of the α-cells into insulin-producing β-cells was described in mice where β-cells were ablated [86,87]. It was also showed that insulin-producing cells can be generated from the adult duct cells by glucagon-like peptide-1 (GLP-1) treatment [88]. The presence of PPCs was confirmed in the rats and humans duct, and the differentiation potential of those cells was evaluated [89,90]. Additionally, acinar cells have been proved to be able to transdifferentiate into β-cells in vivo and in vitro with a generation of the duct cells as in-between step [91,92,93,94,95].

## 6. Induced Pluripotent Stem Cells (iPSC)

**Figure 1:**
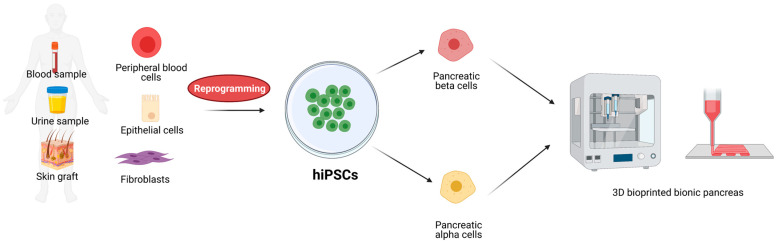
Schematic representation of iPSCs sources, possible differentiation, and application for 3D bioprinting of bionic pancreas. Created with BioRender.com accessed on 17 June 2021.

In 2006, Kazutoshi Takahashi and Shinya Yamanaka have published crucial results presenting a defined group of four transcription factors (TF), Oct3/4, Sox2, Klf4, and c-Myc (OSKM, Yamanaka factors), which overexpressed in embryonic and adult fibroblasts restored their pluripotency [96]. Since then, the protocol to obtain reprogrammed cells with stem cell-like morphology, potency, and expression profile called induced pluripotent stem cells (iPSC) have been widely implemented and developed [50,97,98,99,100]. Since they can be obtained from any cell type iPSCs have become of great interest as theoretically unlimited sources of stem cells. Human iPSCs (hiPSC) derived from easily accessible cell sources such as skin graft [101], peripheral blood [102], or urine [103] can be considered as an advantageous alternative of stem cells applied in regenerative medicine due to minimally invasive collection from the patient and autografted without immunomodulation (Figure 1). Moreover, hiPSC-based therapy does not raise ethical questions concerning the source of stem cells. Since 2006, the protocol of iPSC derivation has been extensively improved. One of the basic concerns was potential tumorigenicity caused by the uncontrolled activity of Klf4 and c-Myc which are defined as proto-oncogenes [97,104]. Across years the number and composition of TF cocktails have been modified including the addition of other proteins such as Nanog, Lin28, and Esrrb [105,106,107] and reduction to only two or even one component [108,109,110,111]. Although these manipulations aimed to affect expression as little as possible, another problem to tackle was the efficient delivery of exogenic factors and their repercussions. Initially, transformed cells were transduced with lenti- and retroviruses carrying open reading frames for particular factors [97,101]. Such approach raised the risk of spontaneous integration of viral DNA to the cellular genome which, in turn, could lead to increased oncogenic activity, especially in the region of the c-Myc gene [104,112]. To circumvent this, novel transformation strategies have been developed based on non-integrating viruses, small molecules, or synthetic mRNA. The first wave of improved solutions has offered a variety of viral-origin non-integrating DNA delivery approaches including PiggyBac transposon/transposase system with self-excision activity [113,114], non-integrating Sendai virus carriers [115,116], and Epstein-Barr virus-derived episomal DNA plasmid replication [117,118]. Another approach emerged from techniques of in vitro transcription and assumes introduction to the cell mRNA of protein of interest [119,120]. Although the techniques of non-integrating DNA delivery characterize with high efficiency they have been shown to increase the risk of chromosomal aberrations during cell division. On the other hand, the mRNA delivery approach providing no interaction with the host’s genome, therefore, less invasive revealed efficiency below 30% and is time-consuming [13]. Another promising and extensively developed approach of pluripotency induction is the affection of signal transduction pathways with small molecules. Signaling pathways such as Wnt/β-catenin or MAPK/ERK responsible for cell division, cell cycle control, inhibition of apoptosis [121,122,123,124,125,126], etc., have been shown to affect the cellular state of pluripotency after particular small molecule cocktail treatment including, among others, vitamin C, valproic acid, GSK-3 inhibitor, or 5-aza-2-deoxycytidine [127,128,129,130,131]. Protocols for small molecules-based iPSCs are constantly upgraded and modified as dozens of molecules have the potential to modulate essential signaling pathways. As an example, in the work of Zhao and colleagues, the authors presented a 1000-fold more efficient iPSC protocol by adding four compounds to a seven-component small molecule cocktail established two years earlier [131,132]. Although delivery of small compounds is much more efficient, broad knowledge about the role of particular signaling pathways in cellular processes and potential off-targeting of tested compounds is pivotal.

## 7. Available β-Cell Differentiation Protocols

**Figure 2:**
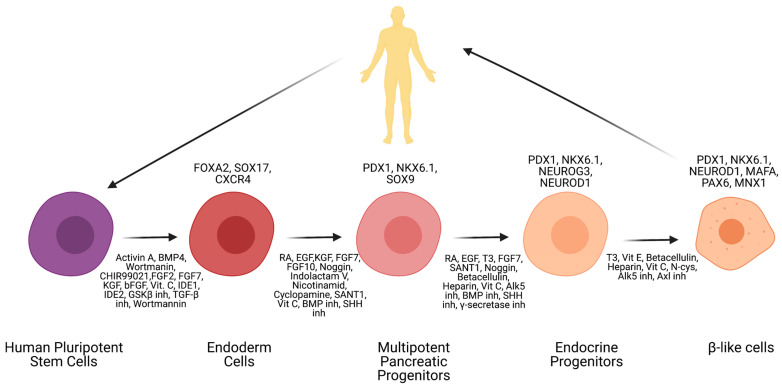
Schematic representation of stem cells differentiation protocol into β-like cells. Created with BioRender.com accessed on 17 June 2021.

Currently, scientists are highly focused on establishing the perfect differentiation protocol that will allow the transformation of hiPSCs or hESCs into functional insulin-producing cells. Thanks to this interest, several protocols have been established until now. In Table 1, there is a summary of the most commonly used protocols [15]. According to available literature, the differentiation of hPSCs into β-like cells could be achieved by the application of listed below growth factors and small molecules [133,134,135,136,137,138,139] (Figure 2). First, by using various growth factors (activin A, BMP4, Wortmanin, CHIR99021, FGF2, FGF7, KGF, bFGF, Vit C, IDE1, IDE2, GSK3β inh, TGF-β inh, Wortmannin) hESCs or hiPSCs are differentiated into definitive endoderm cells. This step can be confirmed by analysis of major markers—FOXA2, SOX17, and CXCR4. The next step is transformation into multipotent pancreatic progenitors (growth factors: RA, EGF, KGF, FGF7, FGF10, Noggin, Indolactam V, Nicotinamide, Cyclopamine, SANT1, Vit C, BMP inh, SHH inh). This step is confirmed by markers PDX1, NKX6.1, and SOX9. The second to last step is transformation into endocrine progenitors with the use of growth factors (RA, EGF, T3, FGF7, SANT1, Noggin, Betacellulin, Heparin, Vit C, Alk5 inh, BMP inh, SHH inh, γ-secretase inh). It can be deemed successful if markers PDX1, NKX6.1, NEUROG3, and NEUROD1 are distinguishable. The last step is creating maturing β-like cells with the use of T3, Vit E, Betacellulin, Heparin, Vit C, N-cys, Alk5 inh, and Axl inh. β-like cells are achieved if markers such as PDX1, NKX6.1, NEUROD1, MAFA, PAX6, MNX1, and Insulin are present.

The first systematized method allowing for differentiation into insulin-producing cells was introduced by Zhang et al. in 2009 [140]. It involved both, hESCs, and hiPSCs and the authors managed to establish cultures of mature pancreatic insulin-producing cells. The protocol consisted of 4 major steps, as the review by Cierpka-Kmiec et al. suggests, which go in order: (1) Endoderm induction (Activin A, Wortmannin); (2) Pancreatic specialization (Retinoic acid, FGF7, NOGGIN); (3) Progenitor expansion (EGF); and (4) Maturation (bFGF, Exendin-4, BMP4). Every step was confirmed by proper expression markers, in order: (1) Sox17, Foxa2; (2) Pdx1, Pax1, Pax6, Hnf6; (3) Pdx1, Foxa2, Sox9, Hnf1B; (4) MafA, insulin, Glut2, Nkx6-1, Glucokinase Tcf1. In 2014, Rezania et al. [141] created a more concise and detailed method, which was based on previously reported one and it involved hESCs. The main difference was the number of steps taken during differentiation, as it amounted to 7 main stages: definitive endoderm, primitive gut tube, posterior foregut, pancreatic endoderm, pancreatic endocrine precursors, immature β-cells, and maturing β-cells. In this protocol, researchers did not use Activin A in any of the steps, instead, they relied on GDF8, and CHIR-99021 during the transformation into definitive endoderm. In later steps, they implemented (among others) Ascorbic acid (Vit C), FGF7, SANT-1, Retinoic acid, LDN193189, and at the end T3, ALK5 inhibitor II and XXiI. The same approach was used by Petersen et al. in 2017 [145]. Additionally, in 2014, another group of scientists designed a protocol for the transformation of hPSCs into stem cell-derived β-cells [133]. Their goal was to create a strategy for the large-scale production of functional human β-cells from hPSCs in vitro. They managed to achieve this by comparing and designing a 6-step protocol which is a modification of a standard protocol and it takes place in a three-dimensional cell culture system. Growth factors used involved Activin A, CHIR99021, KGF, SANT-1, Retinoic Acid, LDN193189, XXI, ALK5 inhibitor II, and T3. Its stages and growth factors prove it to be a mash-up of protocols described above. Other protocols that describe the transformation of stem cells into insulin-producing cells are variations of the ones mentioned in detail in earlier sections of this review. They usually differ slightly in the concentration of added growth factors or describe a different number of stages (though with highly similar growth factor additions and medium exchanges [134,146,147,148]. Interesting advancement was implemented by Nair et al. 2019 [142]. Although using a quite sophisticated process, divided indirectly into 10 steps, they performed isolation, sorting, and reaggregation of cells on day 20 of culturing and transformation to allow clustering of immature β-like cells. This allowed them to create islet-sized enriched β-clusters (eBC). Li et al. 2020 modified the existing protocols for application in the H9 hESC line and proposed β-like cells population enrichment by sorting only CD9^−^ cells, which are characterized by higher insulin production and spontaneous formation of islets like organoids [143]. Another interesting approach was applied by Yoshihara et al. 2020. They identified that non-canonical WTN4 signaling is important for the maturation of β-like cells, which after generation of human islet-like organoids, and transplantation into diabetic mice were able to restore the glucose homeostasis [144].

## 8. Challenges in Obtaining Fully Maturated β-Cells

All described above protocols lead to obtaining β-like cells which are able to secrete insulin and C-peptide in vitro. Nevertheless, very often they display immature β-cells characteristics, such as co-expression of insulin/C-peptide and glucagon or somatostatin [149,150,151] and low levels of insulin/C-peptide secretion [152,153]. Usually, just a small fraction of β-like cells show calcium response to glucose, which is usually slower and lower compared to adult islets [133,134,141]. Frequently, they are also not able to terminate calcium flux when glucose is not present, and they are not able to quickly secrete insulin in dynamic perfusion assay [141]. Another drawback is that transplanted β-like cells, in vivo can secrete insulin only after 2-6 weeks post-transplantation whereas the human islets are able to do it immediately [133,134,141]. Another approach taken by scientists is in vivo maturation of the pancreatic endoderm or pancreatic progenitors to mature β-cells. It was shown that cells transplanted into immunodeficient mice or rats were able to differentiate and maturate into pancreas β-cells which were able to secrete C-peptide in response to meal or glucose challenge [154,155,156]. The big drawback of this approach is the long time that is needed to reach maturation by transplanted cells. So far this approach was only explored in mice and rats, and even for those two closely related species differences in the fate choice between pancreatic endocrine and exocrine cells and α and β cell lineages were significant [156]. Considering that the maturation environment may differ even among human recipients probably is better to focus more on the in vitro differentiation to be sure what kind of the cell population is transplanted into a patient and to be able to reach normoglycemia immediately after transplant as it takes place during human islets transplantation. Despite those drawbacks, company ViaCyte performed two clinical studies where hESC pancreatic progenitors [154,157] were transplanted into T1D patients (NCT02239354, NCT03162926). The main difference between the two trials was applying different encapsulation devices and using modified membranes, which seems to be crucial for successful treatment. Results obtained during second clinical studies showed that transplanted cells secrete C-peptide and positive results were reported for 30% of the patients [158]. In the future, companies plan to further optimize encapsulation strategies.

β-like cells obtained from hESC by Zhang et al. 2009 were mono-hormonal, but just around 25% of obtained cells were producing insulin and expressing PDX1 [140]. Cells were also able to respond to the KCl and glucose stimulation by secretion of C-peptide [140]. According to the protocol proposed by Rezania et al. 2014 also mono-hormonal β-like cells were obtained. Additionally, to PDX1 and NKX6.1, they were showing MAFA expression, which is important for β-cells maturation [159]. Nevertheless, glucose-stimulated insulin secretion displayed by those cells was delayed and slower compared to human islets what suggest that obtained cells are functionally immature comparing to the human β-cells. However, when cells were transplanted to immunodeficient STZ-diabetic mice they were able to restore normal glucose level [141]. Pagliuca et al. 2014 in their protocol obtained stem cells-derived β-cells (SC-β cells), which were expressing markers like adult β-cells, but there were still differences in the expression level. SC-β cells were able to flux Ca^2+^ in response to glucose to a similar extent to mature β-cells and were able to package insulin into secretory granules. Additionally, cells transplanted to the diabetic mice were able to secrete insulin but on a lower level than human islets [133]. Nair et al. 2019 after modification of their previous protocol [134] managed to generate enriched β-clusters (eBC) from immature β-like cells [142]. They showed that the clustering of β-like cells has a positive impact on their maturation [160]. eBCs showed a response to glucose similar to human islets even though on the lower level. A significant and rapid increase in calcium influx after stimulation with high glucose and KCl was observed and what is also relevant the flux returned to baseline after glucose concentration was lowered. Mitochondria morphology of the eBCs was also different with the increase in the mitochondrial mass compared to the level observed for β-like cells. Another improvement was also noticed after eBCs transplantation into mice when C-peptide secretion could be detected as short as three days post-transplantation and was maintained for a long time [142]. In their protocol, Yoshihara et al. 2020 proposed activation of non-canonical WNT signaling as beneficial for metabolic maturation of islet-like organoids (HILO) obtained from differentiated β-like cells. After transplantation into diabetic NOD/SCID mice, HILOs were able to restore glucose homeostasis for more than six weeks. In HILOs, an increase in mitochondrial content was also observed, which suggested improvement in cells maturation [144]. Most likely delivery of the β-cells as clusters similar to human pancreatic islets is the most promising approach. As it was shown that it gives the best results in the case of the maturation of β-like cells, and allows to reach normoglycemia in a short time, as it takes place after cadaveric islets transplantation.

The first animal trials were also performed with the application of SC-derived islets or islet-like organoids. It was shown that conformal-coated human SC-derived islets were able to reverse diabetes in mice [161]. Promising results were also obtained with polymer encapsulated human SC-derived β-cells in mice [162]. Pre-clinical studies were also performed in the non-human primates by Vertex Farmaceuticals. Used islets-like organoids were able to decrease insulin intake by 60% [163].

## 9. Available α-Cell Differentiation Protocols

Generating α-cells from various types of stem cells has not yet gained the same popularity as generating insulin-producing cells. Nevertheless, there are indications that α-cells play role in T1D etiology, especially considering the importance of α-cells β-cells interactions [164,165]. Still, lots of work has been done in this area in the last 10 years. The most famous research was done by Rezania et al. in 2011 [166]. They developed a six-step differentiation protocol that allows conversing human embryonic stem cells into functional glucagon-producing cells. It is based on the native development of the endocrine pancreas. In the first step, they induced stem cell transformation into mesoendoderm by the addition of growth factors (Activin A., Wortmanin, and FGF2) for 3 days. Mesoendoderm cells were then differentiated into endoderm progenitors in the second step (FGF and Cyclopamine-KAAD). Endoderm progenitors were further transformed into foregut progenitors (step three) with the use of Cyclopamine-KAAD, Retinoic acid, FGF7, and NOGGIN as growth factors. In the fourth step, foregut progenitors were differentiated into endocrine precursors by the use of three growth factors (ALK5 inhibitor II, NOGGIN, and DAPT). The fifth step involved transformation into immature endocrine cells with ALK5 inhibitor II. The last step (fourth) consisted of cells maturation into α-cells (in clusters with β-cells) and lasted for 7 days. Every step was confirmed by the expression of proper genetic markers. It was as follows: Mesoendoderm—CXCR4, FOXA2, SOX17; Endoderm progenitors—HNF4α, PDX1; Foregut progenitors—NGN3, HNF4α, PDX1; Endocrine precursors—NEUROD, NGN3, PAX4, PAX6, NKX2.2, ARX; Immature endocrine—GCG, INS, ARX; α-cells—GCG, ARX.

Peterson et al. in 2020 proposed a protocol of differentiation of stem-cells derived α-cells (SC-α) [167]. They characterized two α-cells maturation stages. The first in which cells were showing both insulin and glucagon expression (were further described as pre-α cells) and the second maturation stage, where cells were expressing just glucagon (SC-α cells). They described that lowering expressing the NKX6.1 by removing KGF, SANT-1, and introducing LDN during step four is crucial for redirecting differentiation into α-cells. They showed that pre-α cells are able to maturate to mono-hormonal, glucagon secreting cells both in vitro and in vivo. They described that PDBu addition during step six is important for obtaining SC-α cells. According to this protocol they managed to obtain around 30% of SC-α cells that were secreting glucagon and were able to stop the secretion in response to the glucose.

## 10. Immune-Related Aspects of T1D and Possible Applications of Stem Cells in Therapy

**Figure 3:**
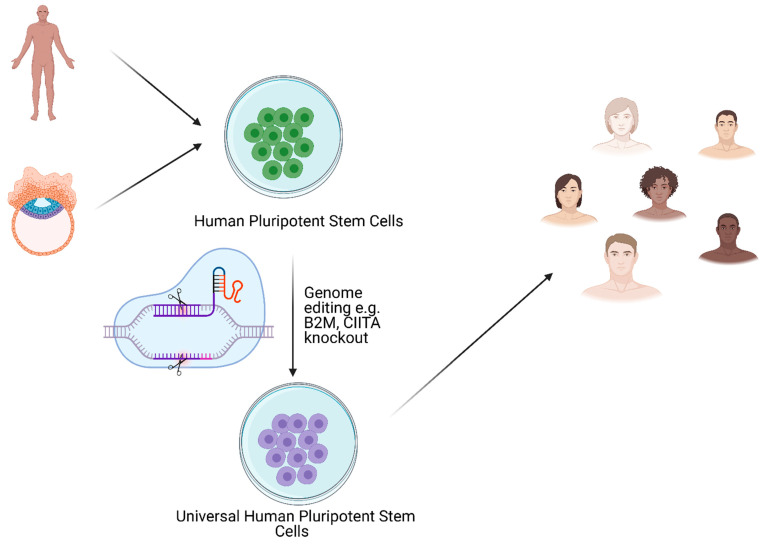
Possible approach for obtaining universal human pluripotent stem cell line. Created with BioRender.com accessed on 17 June 2021.

Although molecular pathomechanism standing behind T1D remains not fully understood, several works point to disturbance in human leukocyte antigen (HLA) system expression in T1D patients [26,168,169]. HLA (or major histocompatibility complex, MHC) is a cluster of surface proteins pivotal in proper recognition of antigens by distinct types of lymphocytes T. All *HLA*-coding genes are located on chromosome 6 and are highly polymorphic which reflects in many variants of particular HLA protein which, in turn, occur in multiple combinations on cells [170,171,172]. Such a complex repertoire ensures flexibility and fine-tuning of the adaptive immune system. However, certain combinations of HLAs have been correlated to a higher risk of appearance of autoimmune disorders, such as T1D [173,174], rheumatoid arthritis [174], coeliac disease [175], etc. According to this knowledge, the cell replacement-based approach of T1D treatment must face the immune barrier issue of whether a patient will receive SC-derived-β cells as either auto- or allograft. In the first case, reprogrammed cells will still be laden with invalid HLA repertoire which will lead to renewed auto-aggression. The second case will require immune suppression to prevent rejection of the graft. A promising solution to both scenarios would be to make transplanted cells immunologically inert. Works of many groups have suggested that such an effect would be possible with the inactivation of HLA genes in cells of interest. Of others, two approaches based on the inactivation of *B2M* and *CIITA* gene have been intensively examined [176,177,178,179,180,181,182,183,184]. *B2M* encodes β-2-microglobulin, a protein that forms heterodimers with HLA Class I proteins present on surface of nearly all nucleated cells in the body and responsible for the presentation of intracellular antigens [170,185,186]. *CIITA* gene encodes the transcription factor which activates expression of HLA Class II proteins presenting extracellular antigens and stimulating indirectly specific antibody-producing B lymphocytes [170,187,188,189]. Altogether, double knock-out of *B2M* and *CIITA* genes leads to disabling of nearly all HLA proteins in the cell. However, some of the published results have led to the conclusion that the inactivation of HLA proteins alone will not be sufficient. Besides antigen presentation, the immune system distinguishes the host’s own cells from “invaders” by the presence of particular protein markers on their surface. A very interesting tool for the creation of universal stem cell lines is genome editing, especially clustered regularly interspaced short palindromic repeats (CRISPR)/CRISPR-associated protein 9 (Cas9) seems to be very promising when it allows, in an easy and efficient way, introduce desired mutations (Figure 3). First attempt to ablate HLA class I with the application of CRISPR/Cas9 was done in hematopoietic stem cells where *B2M* was targeted [180]. In 2017, Hong et al. simultaneously knocked out *HLA-A*, *-B*, and *-C* obtaining similar results as when *B2M* was inactivated [190]. CRISPR/Cas9 was also used to knockout *HLA-B* in iPSCs what resulted in better immunocompatibility and what is important preserved differentiation capacity [191]. Gornalusse et al. in 2017 obtained modified iPSCs by the knockout of the *B2M* gene and overexpression of HLA-E single-chain dimers or trimers, such cells were not recognized by CD8^+^ T-cells and resistant to NK-mediated lysis [192]. In 2019, two groups presented results from stem cells with disrupted HLA proteins and additional overexpression of CD47 protein which is recognized by macrophages as a “don’t-eat-me” signal. Modified cells remained unrecognized by the immune system for a long time after transplantation to mice and retained pluripotent properties [193,194]. In the same year, Xu et al. 2019 performed the knockout of *HLA-A*, *-B*, and *CIITA* in the iPSCs, and at the same time knocked out just one allele of *HLA-C* obtaining the monoallelic line. Non-classical HLA class I (HLA-E, -F, -G) remained unmodified. As a result, obtained cells were not recognized by T cells and NK cells. Authors estimated that around 12 monoallelic HLA-C-retained iPSCs lines would be sufficient to cover more than 90% of the world population [195]. Another approach was used by Lei Shi et al. 2020, where *B2M* was knocked out with CRISPR/Cas9 what allowed to avoid T-cell-directed immune response and at the same time overexpression of Beta2m-HLA-G1 fusion protein was performed what caused inhibition of NK cells mediated cytolysis [196]. Such combined manipulation would be a suitable solution in cell-replacement approaches for T1D. On the other hand, as transformed cells become out of any organismal control, it would raise an extremely challenging long-term safety issue. It has been previously shown that iPSCs may represent a tendency to tumorigenicity due to point mutations occurring as a consequence of invalid epigenetic reprogramming of cells [197,198]. Additionally, re-differentiated iPSC may not fully undergo differentiation or uncontrollably reverse the differentiation fate and turn back into a pluripotent state followed by teratoma formation [199,200]. All these potential risks give rise to a high need to provide an efficient self-control tool and an inducible safety switch that enables controlled apoptosis in case of emergency. There are known several approaches based on the suicide gene technique. Literally, it assumes the introduction of a gene variant that expresses a protein prone to the exogenous agent to the cell genome. Since 2000, strategies based on CD20 overexpression [201], ganciclovir-metabolizing HSV-TK viral kinase [202], or proapoptotic caspase-9 with a caspase-recruiting domain substituted to mutated drug-binding domain from the human FK506-binding protein (iCASP9) sensitive to rimiducid (AP1903) [203,204] have been presented. Although the two first approaches may exhibit side effects caused by off-targeting of anti-CD20 antibody to native CD20-positive cell populations or required instant ganciclovir treatment of herpes simplex virus infection, respectively, the latter approach is thought to be promising as several works have shown [205,206,207]. Recently, the iCASP9 system has been improved by precise, targeted location in the genome in intron 1 of the *PPP1R12C* gene (AAVS1 locus) [208]. The AAVS1 locus is well characterized, and gene editing of this region has been shown to not cause any aberrations in self-renewal or pluripotency of hESCs and iPSCs [209,210,211]. An approach worth considering would be incorporation into the genetically stable region (i.e., aforementioned AAVS1 locus), a cassette encoding apoptotic trigger by which expression would be conditioned by the expression of a particular oncogenic marker. As such, a considerable construct could be composed of an open reading frame for P53 protein, a well-described pro-apoptotic factor, under promoter inhibited by repressor protein. In turn, the transcript of the repressor could be comprised with binding sites for microRNA-21 by which expression has been shown to be significantly elevated in many types of tumors. Such a negative feedback loop could allow the transformed cells to initiate programmed death as an only uncontrolled expression of oncogenic marker occurs.

## 11. Artificial Islets and 3D Bioprinted Pancreas for T1D Treatment

Producing α- and β-cells from any kind of stem cells will not be a full solution for the treatment of diabetes. Recent works suggest that interplay between cells within pancreatic islets plays a role in the proper reaction for glucose stimuli [212,213,214,215,216,217,218]. Cells within a 3D structure responded much better to the change of glucose level in GSIS tests. Currently, there are attempts to develop functional in vitro models of pancreatic islets in which the cellular microenvironment is fully preserved [219,220,221]. Using the issues of microflow and biomaterial engineering, the possibility of culturing and monitoring cells in a permeable 3D microenvironment was demonstrated. Combining different types of cells with each other, which are then suspended in biologically appropriate protein hydrogels, allows the formation of spatial tissue systems. In such models, cells interact with each other and the protein hydrogel, which is their barrier, is also a representation of their native environment [222]. In the case of pancreatic islets, attention should be paid to the islets of the pancreas are composed of different types of endocrine cells, their micro-vascularization is also important, which is crucial for adequate glucose homeostasis [223,224]. The microfluidic method seems to be the most appropriate and precise method for the encapsulation of single cells in biological hydrogels having the ability to cross-link under given physical and chemical conditions [225,226,227]. An important aspect of such a technological process is the possibility of optimizing the encapsulation parameters (shell thickness, hydrogel porosity), which can be modified in terms of selective permeability, as well as the size of the micro-organ produced and the number of cells used to produce them, which is extremely important when trying to create micro-vascularization [219,228]. Those parameters are usually optimized in the lab scale, the next challenge is switching to the industrial scale. The 3D bioprinting can help to overcome some of those obstacles, because of the proper distribution of the cells in the scaffold and the hypoxia improved by the vascularization [229].

Reproducing of islets microenvironment and producing even “perfect artificial islets” will not solve the problem of diabetes as transplantation of pancreatic islets is at the crossroads due to imperfect transplantation results. It is unlikely to think that the transplantation of artificial islets will produce better results. If we want to recreate the natural environment for islets with extracellular matrix and vasculature, it seems that the 3D bioprinting technique may be a solution for that problems, while it allows creating organs in fully controlled in vitro conditions. This technology allows bioprinting with the viable cells obtained from the cell cultures. Many aspects should be optimized to obtain a functional bionic organ. The first important aspect is the choice of proper bioprinting method. Nowadays, four main bioprinting methods are available: micro-extrusion [230], inkjet [231], laser [232], and stereolithography (SLA) [233]. The most popular in the field of tissue engineering are currently extrusion-based methods and SLA [234]. The other important aspects which should be thoughtfully evaluated are, the cell’s concentration in the bioink and proper bioprinting conditions, like pressure, printing speed, or crosslinking method [234]. So far there are not many published reports describing applications of 3D bioprinting for T1D treatment. In the first try, Marchioli et al. successfully used rat β-cell line and human, and mice islets to print in the designed 3D scaffold applying alginate-based bioinks [235]. They did not report changes in the cell’s viability or morphology. Additionally, rat islets were printed with the application of 3D bioprinting technology, the impact on the cells viability, morphology, and functionality was small [236]. In 2019, the Polish team lead by M. Wszoła 3D bioprinted a bionic pancreas with the full vasculature using pancreatic islets, bioinks with the extracellular matrix, and endothelial cells which had a diameter of 3 × 3, 5 × 5 cm^3^ and had 600,000 islets equivalents, which produced insulin (patent proceeding, manuscript in preparation). The decellularized extracellular matrix obtained from the pig’s pancreas was used for the preparation of bioinks. In the obtained product detergent was not detected, a low level of lipids was present, and high content of collagen was shown [237]. Evaluation of the bioprinting conditions was performed and the right ratio of the islets to bioink was estimated, the UV crosslinking time and the pressure applied to extrude the bioink’s fiber was assessed. The islets inside the obtained bionic pancreas were viable and their functionality was proved by the glucose-stimulated insulin secretion assay [238]. Magnetic resonance imaging showed a perfect projection of planned to received results.

Soft tissue engineering in the future could solve the problem of the organ shortage for transplantation. Now several research teams around the world are focused on the investigation of the 3D bioprinting technology with the application of α- and β-cells, pancreatic islets, or whole bioprinted pancreas for T1D treatment. In the future, universal, stem cell-derived α- and β-cells could be a source of the cells for 3D bioprinted pancreas. Such an artificial organ could be transplanted into any T1D patient without the need for immunosuppressive therapy. This could be a perfect solution for the patient with T1D.

At this point, it is worth pointing to the need for a multidisciplinary approach to the subject. Although individual technologies, such as the use of microfluidics to produce 3D biological systems [239,240], the production of protein hydrogels to recreate the native environment for the cells used, or the transformation of stem cells [141,166], and 3D bioprinting technology for recreating 3D structure or organ, as well as biodegradation of tissue systems, are the goal of many scientists, the most optimal approach seems to be to combine all these activities into one technology that allows the creation of functional, neutral for the organism of recipients, bionic pancreas.

## Figures and Tables

**Table 1:** List of available protocols for stem cell differentiation into β-cells.

Authors	Growth Factors and Small Molecules Used in the Study	Outcome
Zhang et al., 2009 [140]	Act A, Wort, RA, Noggin, FGF7, EGF, Nico, bFGF, Ex-4, BMP4	Successful differentiation into mature insulin-positive cells
Rezania et al., 2014 [141]	GDF8, GSK3β inh, FGF7, Vit C, RA, SANT, TPB, LDN, SANT, Alk5 inh, T3, LDN, GS inh XX, N-cys, AXL inh	Approximately 50% of cells were insulin-positive
Pagliuca et al., 2014 [133]	Act A CHIR, KGF, RA, SANT1 LDN, PdbU, T3, XXI, Alk5 inh, Heparin, Betacelluin, CMRL	Around 33% of cells were C-peptide-positive
Russ et al., 2015 [134]	WNT3a, Act A, TGFb inh, KGF, RA, Cyclopamine, Noggin, EGF, TBP, Alk5 inh	Simplified protocol, around 23% C-peptide-positive cells
Nair et al., 2019 [142]	Wnt3a, Act A, TGB inh, KGF, RA, EGF, ALK5 inh, XX inh, LDN, Vit C	Clustering of immature β-like cells as a critical step in gaining full functionality
Li et al., 2020 [143]	Act A, Chir99021, FgF-β, Vit C, KGF, Sant1, RA, Noggin, EGF, RepSox, GC1, LDN, CoE, Y-27632, R428, Trolox, N-cys	Generation of pancreatic like islets that contained approximately 30–40% of β-like cells
Yoshihara et al., 2020 [144]	Act A, GSK3β inh, FGF7, Vit C, RA, TGFβ inh, BMPR inh, Hedgehog inh, PKC, T3, Alk5 inh, Notch inh, Vit C, Vit E, cAMP, WNT4	50–60% of human islets-like organoids cells expressed insulin and β-cells markers
